# Supplementary material for: A versatile reverse genetics platform for SARS-CoV-2 and other positive-strand RNA viruses
Source: Nat Commun. 2021 Jun 8;12:3431. doi: 10.1038/s41467-021-23779-5 (PMC8187723; doi:10.1038/s41467-021-23779-5)
Supplement: Supplementary file 1 — Supplementary Information [file 41467_2021_23779_MOESM1_ESM.pdf]

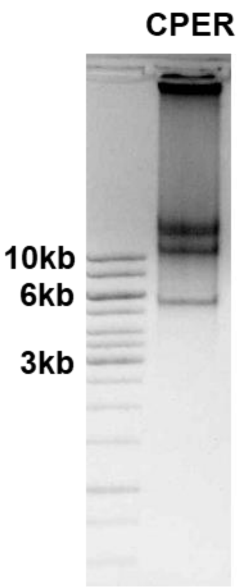

Supplementary Figure 1: Representative CPER gel picture. Gel electrophoresis of CPER mix used for downstream transfection into HEK293T cells. CPER generates circular DNA that could assume supercoiled conformation and form concatemers shown as a smear. This gel is representative of at least three experimental repeats.

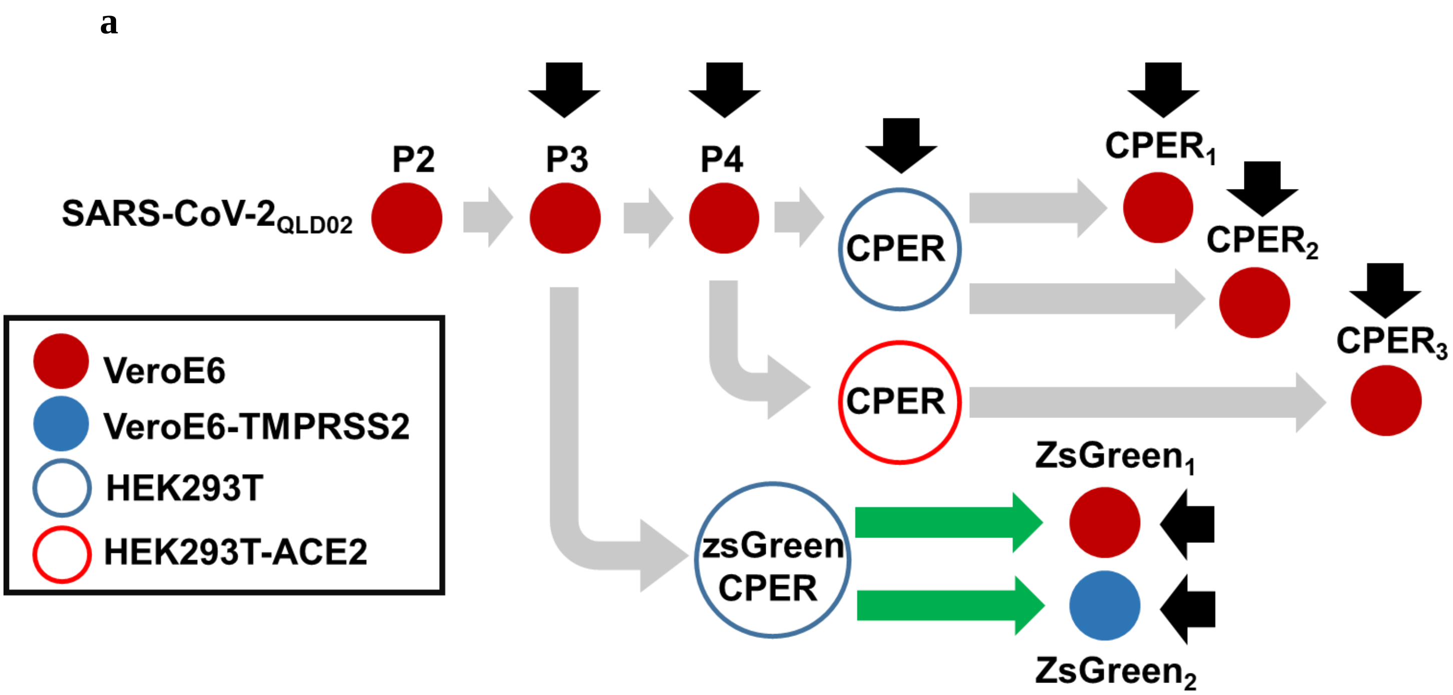

Supplementary Figure 2. Schematic showing the passage history of SARS-CoV-2 isolates and transfection and culturing cell lines used to generate SARS-CoV-2 viruses by CPER. (a) Cell lines used for transfection are shown as empty circles. Coloured in circles indicate cell lines used for virus passaging. Black arrows indicate samples that were deep sequenced using Nanopore.

b

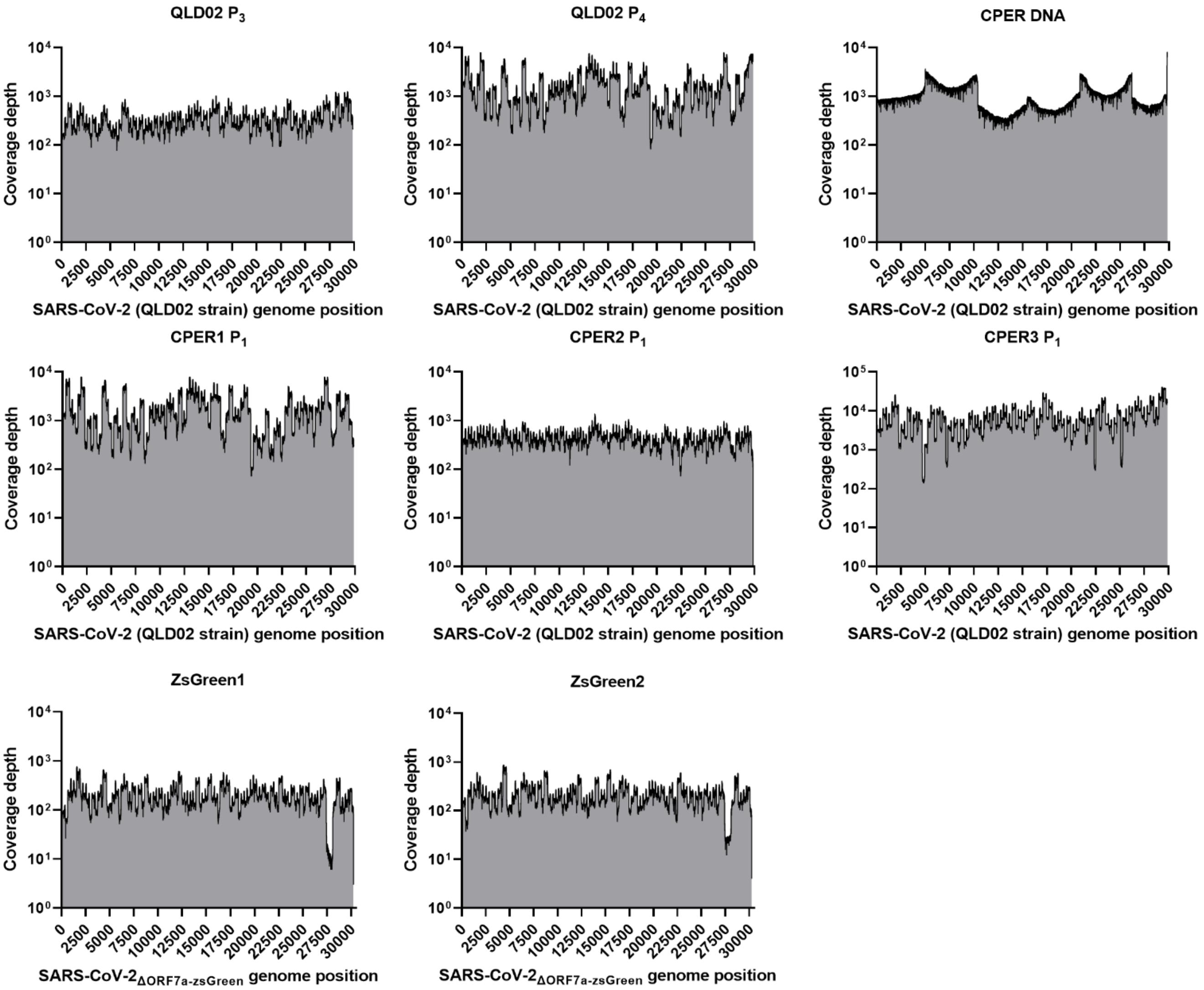

Supplementary Figure 2 (continued). Coverage of SARS-CoV-2 isolates, CPER cDNA and recovered SARS-CoV-2 viruses sequenced using Nanopore MinION. Summary plots of the number of reads in SARS-CoV-2 viruses mapping to the corresponding reference genomes **(b)**, SARS-CoV-2 QLD02 or SARS-CoV-2 zsGreen. Depth of coverage of binary alignment files was determined using samtools depth.

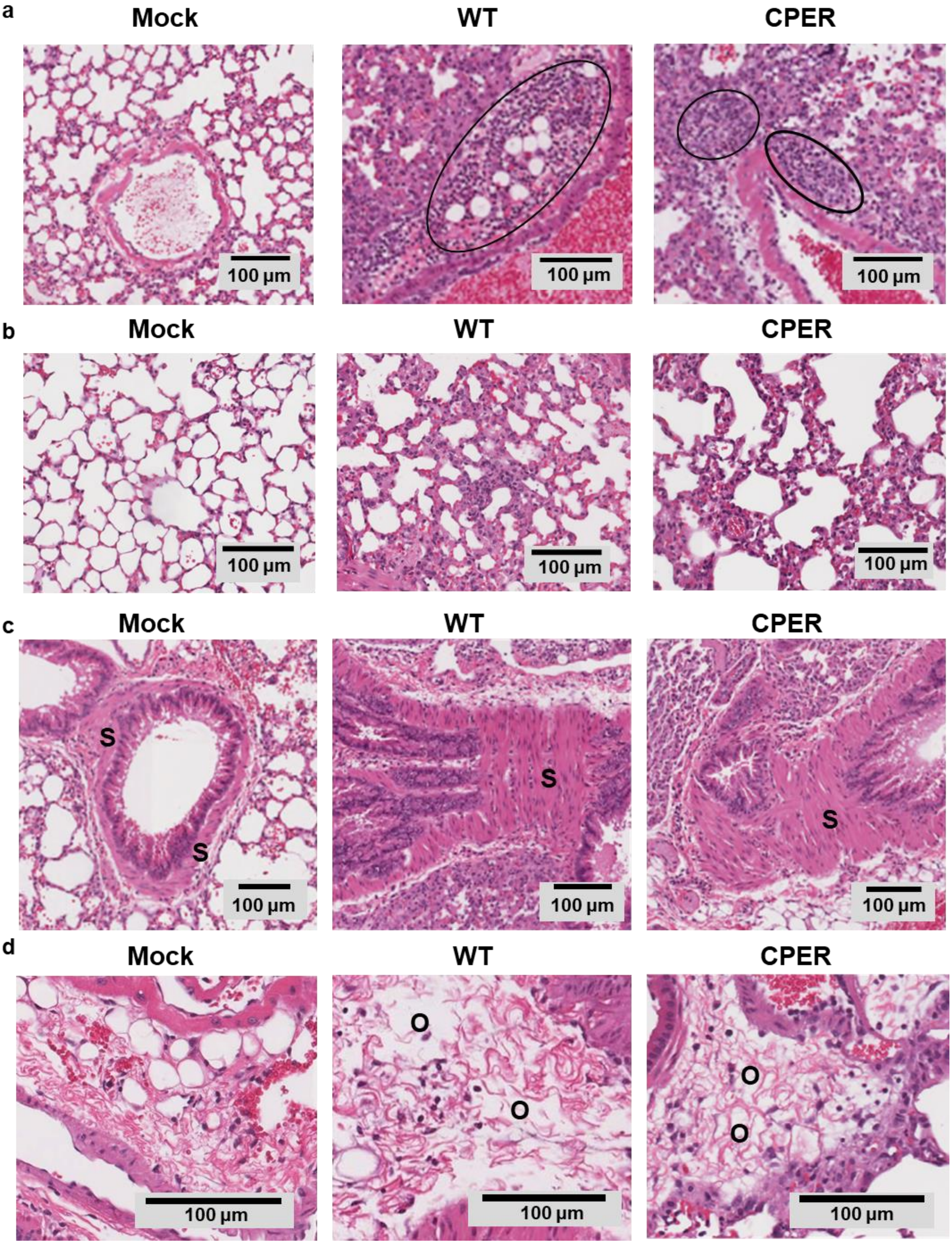

Supplementary Figure 3. Selected histopathological features in lungs from JAX K18 hACE2-transgenic mice infected with wild-type (WT) SARS-CoV-2 isolate and CPER-generated SARS-CoV-2 viruses. Hematoxylin and eosin stained selected lung sections showing: **(a)** mononuclear cell infiltrates indicated by the ellipses, **(b)** thickening of the septa by mononuclear inflammatory cells and interstitial oedema **(c)** smooth muscle (indicated by S) hypertrophy or hyperplasia **(d)** oedema (indicated by **O**) in the eosinophilic collagen bundles. Representative images from **(a-d)** are from three independently analysed samples for each treatment.

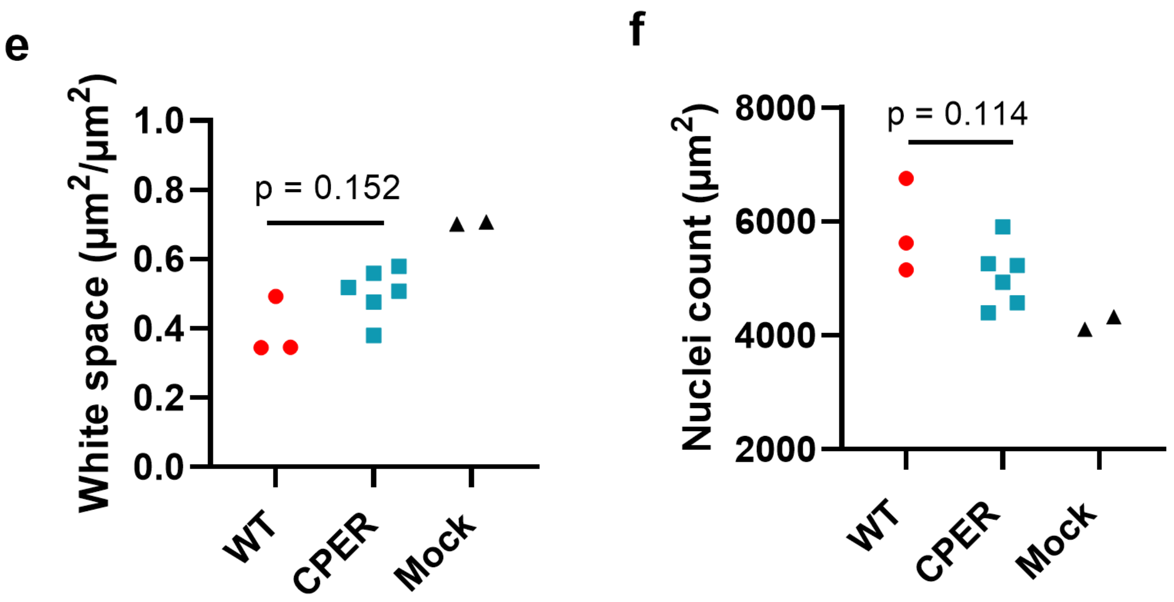

Supplementary Figure 3 (continued). Image analyses. Analyses from two hematoxylin and eosin-stained lung sections per lung were averaged to give one value per mouse for WT virus (QLD02) infected mice (n=3), CPER virus-infected mice (n=6), and mock-infected mice (n=2). Image analyses were undertaken using QuPath v0.2.3. Statistical analyses used two-sample t-tests, assuming unequal variances. **(e)** Image analysis to measure lung consolidation quantitated as areas of white space (unstained air spaces) per μm^2 **(f)** Image analysis of cellular infiltration in lungs quantitated by nuclei count per μm^2. For **(e-f)** WT virus infected mice samples are indicated by red circles, CPER virus-infected mice samples are blue squares and mock-infected mice samples are indicated with black triangles.

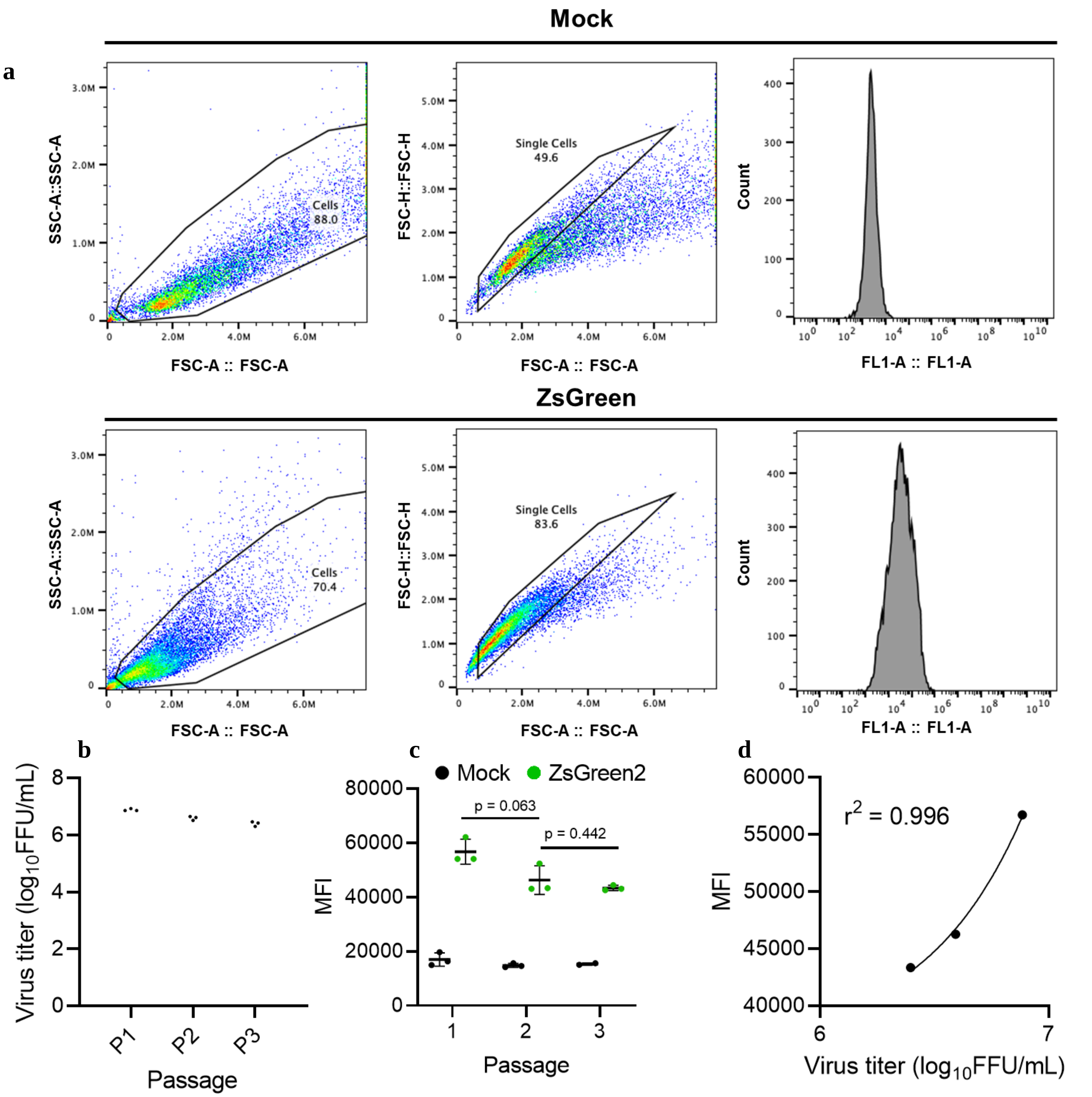

Supplementary Figure 4. Flow cytometry of cells infected with ZsGreen SARS-CoV-2 reporter virus. (a) Mock infected (Mock) and ZsGreen2 infected VeroE6-TMPRSS2 cells were used for gating strategy for flow cytometry analysis. Ten thousand cells were gated using Side Scatter (SSC-A) verses Forward Scatter (FSC-A), single cells were selected using Forward Scatter (FSC-H) vs Forward Scatter (FSC-A). Fluorescence was measured through a 424/44 bandpass filter in front of fluorescence 1 (FL1) detector. Single cells were then analysed for median fluorescence intensity (MFI). Relative fluorescence intensities, which corresponded to relative zsGreen content of gated populations, were acquired on histograms of FL1 pulse area (FL1-A). Flow cytometry was conducted using the BD Accuri6, and analysis performed using the FlowJo V10. **(b)** viral titres of ZsGreen2 virus in supernatants determined by iPA over three passages on VeroE6-TMPRSS2 cells, data shown as individual points from one experiment (n=3) +/- SD. **(c)** MFI of ZsGreen2 infected VeroE6-TMPSS2 cells and Mock infected VeroE6 cells. Statistical analysis was an Unpaired t-test with Welch's correction conducted between ZsGreen2 infected cells from P1-P2 and P2-P3, p-values are two-tailed and shown above comparison. Data shown as individual points from one experiment (n=3) +/- SD. **(d)** a Pearson correlation coefficient between average virus titres from **(b)** (n=3) for each virus passage as determined by iPA and average MFI from **(c)** (n=3) suggests that MFIs of ZsGreen virus correlate with virus titres (r^2 = 0.996).

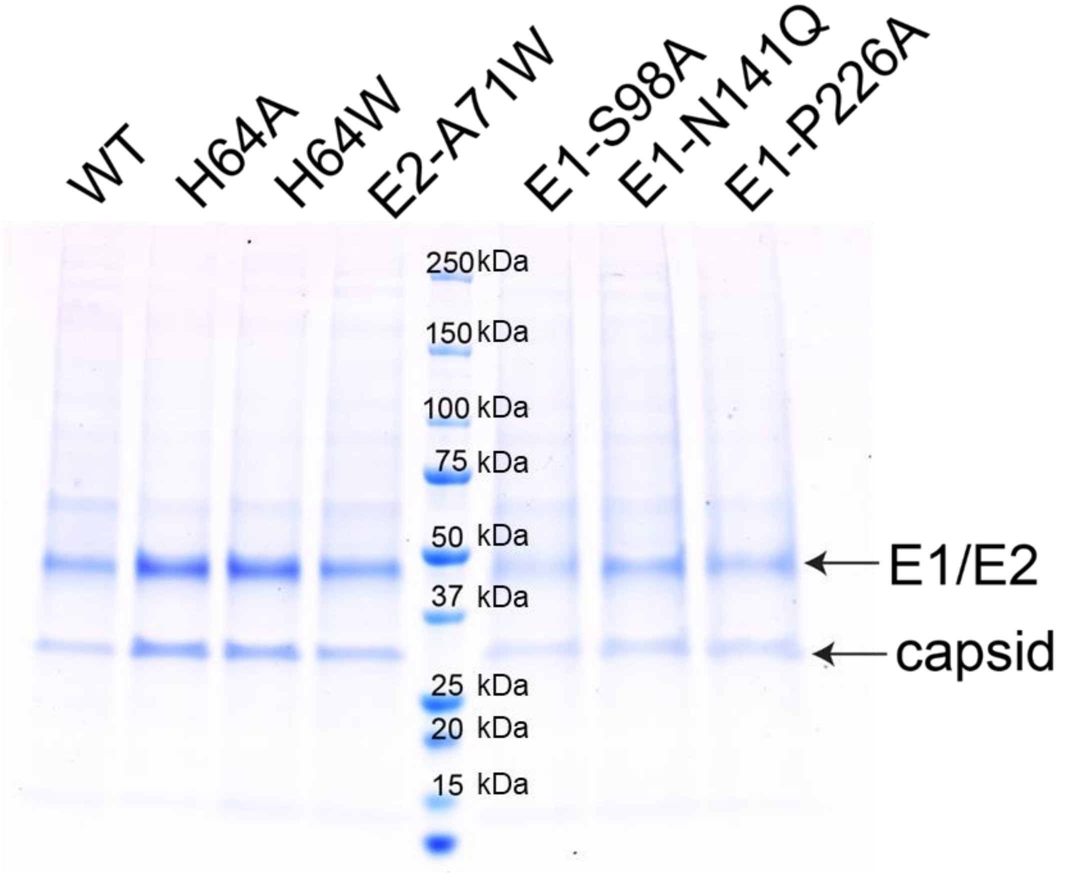

Supplementary Figure 5. SDS-PAGE gel of purified Ross River virus E1/E2 mutants. Gel indicates the three RRV structural proteins E1/E2 and capsid protein from CPER-generated WT virus and CPER-generated RRV mutant viruses. Gel represents a single experiment.

Supplementary Table 1. Percentages of sequencing reads in the parental isolate, CPER cDNA and CPER viruses at variant positions. The following were deep sequenced (i) the parental SARS-CoV-2 QLD02 isolate at passages 3 and 4, (ii) the CPER cDNA fragments amplified from SARS-CoV-2 QLD02 passage 4 cDNA, and (iii) the CPER generated viruses after a single passage in the indicated cells lines. The percentages of sequencing reads for the corresponding nucleotides that code for the indicated amino acids changes in CPER-recovered viruses from the parental SARS-CoV-2 QLD02 isolate sequence are shown.

	QLD02 VeroE6	QLD02 VeroE6	CPER cDNA from P4	$CPER_1$ 293T/ VeroE6	$CPER_2$ 293T/ VeroE6	$CPER_3$ ACE2-293T/ VeroE6
Passage number	P3	P4		P1	P1	P1
SRA Accession number	SRR13883861	SRR13883860	SRR13883867	SRR13883866	SRR13883865	SRR14018671
6207 A to G ORF1ab NSP3 K1981R	1.45%	1.44%	2.18%	0.93%	70.43%	1.16%
6224 C to A ORF1ab_NSP3 H1987N	0.14%	0.44%	0.41%	74.54%	0.56%	0.52%
15709 A to G ORF1ab_NSP12 I5149V	0.55%	1.34%	0.98%	0.90%	74.43%	0.72%
20718 G to A ORF1ab NSP16 M6818I	5.41%	6.78%	5.08%	5.95%	82.45%	2.85%
23607 G to T_Spike_furin_R682L	2.31%	21.81%	27.52%	79.11%	2.84%	93.32%
23607 G to A Spike furin R682G	4.16%	10.23%	10.08%	3.37%	81.64%	1.11%
26270 C to T_E T9I	8.23%	6.81%	4.91%	79.60%	10.93%	5.28%
27303 C to A_ORF6 N34K	1.67%	1.69%	0.75%	72.24%	3.04%	1.89%

Supplementary Table 2. Percentages of sequencing reads in the parental isolate and CPER-generated zsGreen viruses at the furin cleavage site. The following were deep sequenced (i) the parental SARS-CoV-2 QLD02 virus at passage 3, and (ii) the CPER generated zsGreen viruses after 1 passage in the indicated cells. The percentages of sequencing reads for the corresponding nucleotides at the positions 23606-23617 that code for the furin cleavage site are shown.

SARS-CoV-2 Genome position	Nucleotide	Amino acid	QLD02 VeroE6 P3 SRA Accession: SRR13883861	zsGreen 1 293T/ VeroE6 SRA Accession: SRR14018670	zsGreen 2 293T/ VeroE6-TMPRSS2 SRA Accession: SRR14018669
23606	C	R	95.43	91.71	92.37
23607	G		92.59	88.52	88.59
23608	G		89.28	89.19	89.6
23609	C	R	90.17	85.60	88.09
23610	G		84.00	79.50	79.83
23611	G		89.08	90.22	91.72
23612	G	A	94.24	90.97	93.12
23613	C		93.36	89.06	92.96
23614	A		92.38	91.86	95.96
23615	C	R	93.27	93.60	91.86
23616	G		90.86	61.98	80.34
23617	T		93.05	92.91	94.44

Supplementary Table 3. All primers used in this study.

Fragments	Name	Sequences (5' to 3')	Amplicon size (bp)
SARS-CoV-2 CPER F1	CoV-1F	TCCCAGGTAACAAACCAACCAACTTTCG	4995
	CoV-1R	CTTGCGTGTGGAGGTTAATGTTGTCTACTG	
SARS-CoV-2 CPER F2	CoV-2F	CATTAACCTCCACACGCAAGTTGTGGACATG	5394
	CoV-2R	GTCTGTCCTGGTTGAATGCGAACAAACTTATAC	
SARS-CoV-2 CPER F3	CoV-3F	CGCATTCAACCAGGACAGACTTTTTCAGTG	5084
	CoV-3R	GCCACACATGACCATTTCACTCAATACTTGAG	
SARS-CoV-2 CPER F4	CoV-4F	GTGAAATGGTCATGTGTGGCGGTTCACTATATG	5470
	CoV-4R	CCTGGTGCAACTCCTTTATCAGAACCAG	

SARS-CoV-2 CPER F5	CoV-5F	GATAAAGGAGTTGCACCAGGTACAGCTGTTTTAAG	5321
	CoV-5R	GTCGTCGTCGGTTCATCATAAATTGGTTCC	
SARS-CoV-2 CPER F6	CoV-6F	TATGATGAACCGACGACGACTACTAGCG	3689
	CoV-6R	GTCATTCTCCTAAGAAGCTATTAAAATCACATGGGG	
SARS-CoV-2 CPER Linker fragment	CoV-Linker_F	TAGCTTCTTAGGAGAATGACAAAAAAAAAAAAAAAAAAAAAAAAAAAAAAAAAGGGTCGGCATGGC	1119
	CoV-Linker_R	GGTTGGTTTGTTACCTGGGAAGGTATAAACCTTTAATCGGTTCACTAAACGAGCTCTGCT	
SARS-CoV-2 CPER D614G Mutagenesis Mod_Fragment_5A	CoV-5F	GATAAAGGAGTTGCACCAGGTACAGCTGTTTTAAG	2536
	D614G_CPER_R	TGTGCAGTTAACATCCTGATAAAGAACAGCAACC	
SARS-CoV-2 CPER D614G Mutagenesis Mod_Fragment_5B	D614G_CPER_F	ATCAGGATGTTAACTGCACAGAAGTCCCTG	2805
	CoV-5R	GTCGTCGTCGGTTCATCATAAATTGGTTCC	
SARS-CoV-2 D614G Sequencing	D614GSeq_F	ACCAGCAACTGTTTGTGGACC	450
	D614GSeq_R	ATACCTGCACCAATGGGTATGTCAC	
SARS-CoV-2 CPER Mod_Fragment_6A	CoV-6F	TATGATGAACCGACGACGACTACTAGCG	1265
	ORF7-zsGreen_Start_R	TGCTTGGACTGGGCAGCGAGTGTTATCAGT	
SARS-CoV-2 CPER Mod_Fragment_6B	ORF7-zsGreen_Start_F	GGCTCCGCCTTGCCCTTTATAACACTTTGCTTCAC	2168
	CoV-6R	GTCATTCTCCTAAGAAGCTATTAAAATCACATGGGG	
SARS-CoV-2 CPER ORF7_zsGreen	zsGreen_F	ACTGATAACACTCGCTGCCCAGTCCAAGCA	726
	zsGreen_R	GTGAAGCAAAGTGTTATAAAGGGCAAGGCGGAGCC	

RRV CPER F1	RRV-5'UTR-F	GATGGCGGACGTGTGACATCAC	1706
	RRV-NSP1-R	CATTCCTGGGTGTCTCCACTACCC	
RRV CPER F2	RRV-NSP2-F	GGGTAGTGGAGACACCCAGGAATG	2420
	RRV-NSP2-R	GGTCCTACGCACACGGTATGAGG	
RRV CPER F3	RRV-NSP3-F	CCTCATACCGTGTGCGTAGGACC	1639
	RRV-NSP3-R	CCTGGTCCGGTATCAGACGAGAAG	
RRV CPER F4	RRV-NSP4-F	CTTCTCGTCTGATACCGGACCAGG	2892
	RRV-E3-R	GTGCTCTGTTACACTACGGCGG	
RRV CPER F5	RRV-E2-F	CCGCCGTAGTGTAACAGAGCAC	2767
	RRV-E1-R	CCGACGCATTGTTATGCAGGTTACC	
RRV CPER F6	RRV-3'UTR-F	GGTAACCTGCATAACAATGCGTCGG	525
	RRV-3'UTR-R	GACGCCTACGTCCCCGGTGTTATG	
RRV CPER CMV-Alpha-UTR-linker fragment construction	RRV-Linker-no poly A-F	AAAAAAAAAAAAAAAAAAAAAAAAAGGGTCGGCATGGCATCTCCACCTC	1078
	RRV-Linker-no poly A-R	GTGATGTCACACGTCCGCCATCCGGTTCACTAAACGAGCTCTGC	
	RRV_Ultramer	CATAACACCGGGGACGTAGGCGTCTAATTTGTTTTTTAAYATTTTACAAAAAAAAAAAAAAAAAAAAAAAAAAAAAAAAAAAA	134

		AAAAAAAAAAAAAAAAAAAAAAAAAAAAAGGGTCGGCATGG CATCTCCACCTC	
	RRV-PolyA-F	CATAACACCGGGGACGTAGGCGTC	134
	RRV-PolyA-R	GAGGTGGAGATGCCATGCCGACCC	
RRV CPER CMV-Alpha-UTR-linker fragment amplification	Alpha-UTR-Linker-F	GTGATGTCACACGTCCGCCATCCGGTTCACTAAACGAGCTCT GC	1188
	Alpha-UTR-Linker-R	GTGATGTCACACGTCCGCCATCCGGTTCACTAAACGAGCTCT GC	
RRV CPER OpIE2-Alpha-linker fragment construction	RRV-Linker-F	GGGTCGGCATGGCATCTCCACCTC	980
	OpIE2-RRV-R	GTGATGTCACACGTCCGCCATCGTTTACCAGATCGTTGCGGG CTG	
	RRV_Ultramer	CATAACACCGGGGACGTAGGCGTCTAATTTGTTTTTTAAYAT TTTACAAAAAAAAAAAAAAAAAAAAAAAAAAAAAAAAAAAA AAAAAAAAAAAAAAAAAAAAAAAAAAAAAGGGTCGGCATGG CATCTCCACCTC	134
	RRV-PolyA-F	CATAACACCGGGGACGTAGGCGTC	134
	RRV-PolyA-R	GAGGTGGAGATGCCATGCCGACCC	
RRV CPER OpIE2- Alpha-linker fragment amplification	RRV-PolyA-F	CATAACACCGGGGACGTAGGCGTC	1090
	OpIE2-RRV-R	GTGATGTCACACGTCCGCCATCGTTTACCAGATCGTTGCGGG CTG	
RRV CPER E2-H64A mutation	RRV-NSP4-F	CTTCTCGTCTGATACCGGACCAGG	E2-H64A-1 3069
	E2-H64A_R	CGGATCTTTGTGGCGGCGTGGGTAC	
	E2-H64A_F	GTACCCACGCCGCCACAAAGATCCG	E2-H64A-2 2571
	RRV-E1-R	CCGACGCATTGTTATGCAGGTTACC	
RRV CPER E2-H64W mutation	RRV-NSP4-F	CTTCTCGTCTGATACCGGACCAGG	E2-H64W-1 3069
	E2-H64W_R	CGGATCTTTGTCCAGGCGTGGGTAC	
	E2-H64W_F	GTACCCACGCCTGGACAAAGATCCG	E2-H64W-2 2571
	RRV-E1-R	CCGACGCATTGTTATGCAGGTTACC	
RRV CPER E2-A71D mutation	RRV-NSP4-F	CTTCTCGTCTGATACCGGACCAGG	E2-A71D-1 3090
	E2-A71D_R	CCTGAACATCATGACCATCCATATATCGGATC	
	E2-A71D_F	GATCCGATATATGGATGGTCATGATGTTCAGG	E2-A71D-2 2550
	RRV-E1-R	CCGACGCATTGTTATGCAGGTTACC	
RRV CPER E1-N141Q mutation	RRV-E2-F	CCGCCGTAGTGTAACAGAGCAC	E1-N141Q-1 1876
	E1_N141Q_R	CCTCGGTGGTCTGTTGGATGGTGCCATAAC	
	E1_N141Q_F	GTTATGGCACCATCCAACAGACCACCGAGG	E1-N141Q-2 1394
	RRV-3'UTR-R	GACGCCTACGTCCCCGGTGTTATG	
RRV CPER E1-P226A mutation	RRV-E2-F	CCGCCGTAGTGTAACAGAGCAC	E1-P226A-1 2131
	E1-P226A_R	CATGCACAACCCCGGCTGATGGTCTTGAG	
	E1-P226A_F	CTCAAGACCATCAGCCGGGGTTGTGCATG	E1-P226A-2 1139
	RRV-3'UTR-R	GACGCCTACGTCCCCGGTGTTATG	
RRV CPER Mod_Fragment _4 Furin	RRV-NSP4-F	CTTCTCGTCTGATACCGGACCAGG	2853
	RRV-F4.1R	GCATGTCATGGACGCTTCCAGTAGCTC	
RRV CPER Mod_Fragment _5 Furin	RRV-F5.1F	GTAACAGAGCACTTCAATGTGTATAAGGC	2756
	RRV-E1-R	CCGACGCATTGTTATGCAGGTTACC	
RRV CPER Fragment 4 with H5 furin site	RRV-NSP4-F	CTTCTCGTCTGATACCGGACCAGG	2881
	RRV-F4-R.2-H5	CCCCTCTTTTCTTTCTCCGTCTCTCTCTGCATGTCATGGACGC TTCCAGTAGCTC	
	RRV-F5-F.1-H5	GCAGAGAGAGACGGAGAAAGAAAAGAGGGGTAACAGAGCA CTTCAATGTGTATAAGGC	2786

RRV CPER Fragment 5 with H5 furin site	RRV-E1-R	CCGACGCATTGTTATGCAGGTTACC	
RRV CPER Fragment 4 with SFV furin site	RRV-NSP4-F	CTTCTCGTCTGATACCGGACCAGG	2878
	RRV-F4-R.2-SFV	CGACGGTTCGTCGATGGCGTGTTCCGCATGTCATGGACGCTT CCAGTAGCTC	
RRV CPER Fragment 5 with SFV furin site	RRV-F5-F.1-SFV	GCGGAACACGCCATCGACGAACCGTCGTAACAGAGCACTTC AATGTGTATAAGGC	2783
	RRV-E1-R	CCGACGCATTGTTATGCAGGTTACC	

MNV CPER_F1	MNV_F1.F	GTGAAATGAGGATGGCAACGCCATCTTC	2398
	MNV_F1.R	CTGACACAGTTGACAAGTGTTTTGGGC	
MNV CPER_F2	MNV_F2.F	GCCCAAAACACTTGTCAACTGTGTCAG	2697
	MNV_F2.R	CACTCATCCTCATTCACAAAGACTGCTGAG	
MNV CPER_F3	MNV_F3.F	CTCAGCAGTCTTTGTGAATGAGGATGAGTG	2392
	MNV_F3.R	GCAGTAAGCAGAAATCATTTTCACAAAAGGTTTC	
MNV CPER linker fragment construction	pUC19flavi_F	GGGTCGGCATGGCATCTCCACCTCC	1061
	CMV_MNV_R	AGAAGATGGCGTTGCCATCCTCATTTC ACCGGTTCACTAAACGAGCTCTGCTTA	
	NoV_Ultramer	GAAACCTTTTGTGAAAATGATTTCTGCTTA CTGCTTTCTTTTCTTTGTGGTAGTTAGATGC ATTTTAAAAAAAAAAAAAAAAAAAAAAAAAA AAAAAGGGTCGGCATGGCATCTCCACCTCC	121
MNV CPER linker fragment amplification	Ultramer_F	GAAACCTTTTGTGAAAATGATTTCTGCTTACTG	1156
	MNV_Link.R	GAAGATGGCGTTGCCATCCTCATTTCAC	
HuNoV CPER_F1	HuNoV_F1.F	GTGAATGAAGATGGCCTCTAACGACGC	2632
	HuNoV_F1.R	GCCCTCAGTTTTGATGTCGTCGGATG	
HuNoV CPER_F2	HuNoV_F2.F	CATCCGACGACATCAAAACTGAGGGC	2400
	HuNoV_F2.R	CTTGTCTGGGCACGTAAAAATCCATGCC	
HuNoV CPER_F3	HuNoV_F3.F	GGCATGGATTTTTACGTGCCCAGACAAG	2582
	HuNoV_F3.R	AAAAGACACTAAAGAAAAAGAAAG ATAATCAATTTTGTC	
HuNoV CPER linker fragment	HuNoV_ Linker.F	GACAAAATTGATTATCTTTCTTTTTCTTTAGTGTCTT TTAAAAAAAAAAAAAAAAAAAAAAAAAAAAAAGG	1126
	HuNoV_ Linker.R	GCGTCGTTAGAGGCCATCTTCATTCAC CGGTTCACTAAACG	
HuNoV_qPCR	NKP2F	ATGTTYAGRTGGATGAGATTCTC	88
	NKP2R	TCGACGCCATCTTCATTCAC	

CASV CPER F1	CASV 5' linker F	GCAACGATCTGGTAAACACTAAAGAAAATTCTGTTTTCACC C	5229
	CASV F1 R	GGATCATAGGCAATATTAGCATTTGTAGTATTTGTCAAATG C	
CASV CPER F2	CASV F2 F	GCATTTGACAAATACTACAAATGCTAATATTGCCTATGATC C	5181
	CASV F2 R	GCATAGTTTGTTACGTAATTCTGGTTCTAGGTGG	
CASV CPER F3	CASV F3 F	CCACCTAGAACCAGAATTACGTAACAAACTATGC	5279
	CASV F3 R	GGGCTGGCATTTTGAGTTTATCATATATAGATTAGCTAGG	
CASV CPER F4	CASV ORF2a F	CCTAGCTAATCTATATATGATAAACTCAAAATGCCAGCCC	2728
	CASV ORF2a R	GATAGTAGTAGTAGTTTATTACAAAATTCGTAAAAATTTTA GG	
CASV CPER F5	CASV F5 F	CCTAAAATTTTTACGAATTTTGTAATAAACTACTACTACTAT C	1761
	CASV 3'UTR R2	CCAATCGCCATACTACTAAGTCCTAACCGATTTCG	

CASV CPER OpIE2-linker fragment construction	HDVr_F	GGGTCGGCATGGCATCTCCACCTCCTCGCG	979
	CASV 5' linker R	GGGTGAAAACAGAATTTTCTTTAGTGTTTACCAGATCGTTGC	
	CASV Ultramer	TCGGTTAGGACTTAGTAGTATGGCGATTGGCATTACAGGTAAAAAAAAAAAAAAAAAAAAAAAAAAAAAAAAAAAAAAAAAAAAAAAAAAAA AGGGTCGGCATGGCATCTCCACCTC	117
	CASV 3'UTR poly AF2	TCGGTTAGGACTTAGTAGTATGGCGATTGG	117
	RRV-Poly A-R	GAGGTGGAGATGCCATGCCGACCC	
CASV CPER OpIE2 linker fragment amplification	CASV 3'UTR poly AF2	TCGGTTAGGACTTAGTAGTATGGCGATTGG	1072
	CASV 5' linker R	GGGTGAAAACAGAATTTTCTTTAGTGTTTACCAGATCGTTGC	

Supplementary Note 1

Generation of TMPRSS2-expressing Vero E6 cell line

A codon-optimised human TMPRSS2 synthetic DNA fragment was cloned into pLJM1-eGFP plasmid (Addgene # 19319) in place of the eGFP gene. HEK-293T cells were seeded in 6-well plates at a density of 5 x 10^5 cells per well a day before transfection. Transfection was performed using Lipofectamine LTX and Plus Reagent (Life Technologies). Briefly, 150 µl of OptiMEM (Life Technologies) and 10 µl of LTX were mixed and incubated at room temperature for 5 minutes. In a second tube, 150 µL of OptiMEM was mixed with 1 µg of Lenti-TMPRSS2 or pLJM1-EGFP as a control, 0.75 µg of psPAX2 plasmid (Addgene # 12260), 0.5 µg of VSV-G plasmid (Addgene # 8454) and 2.5 µL of Plus Reagent. Both tubes were mixed and incubated at room temperature for 30 minutes. Cell media was removed, and the transfection mixture was added to cell monolayer followed by adding additional 300 µL of OptiMEM. 6 hours post-transfection, the cells were supplemented with 1.4 mL of DMEM with 10% FCS, 1% penicillin/streptomycin and 1% Glutamax (D10) and incubated at 37 °C with 5% CO_2. At 60 – 72 hours post-transfection, the supernatant containing lentivirus was removed, clarified remove cell debris, filtered through a 0.45 µM PVDF syringe filter unit (Millex-HV; Merck), aliquoted and stored at -80 °C.

For transduction, Vero E6 cells were seeded in 6-well plates at a density of 5 x 10^5 cells per well a day before transduction. Media was removed, and 2 mL of lentiviral supernatant was added to cells and cells were incubated for 24 – 26 hours in a 37 °C incubator with 5% CO_2. Following transduction, the supernatant was removed, and new D10 supplemented with 30 µg/mL of puromycin was added. Successful lentiviral

transduction was validated by eGFP fluorescence in a control well. Transduced Vero E6-TMPRSS2 cells were propagated for at least 4 passages in D10 supplemented with 30 µg/mL of puromycin.

For TMPRSS2 immunoblot, cells were seeded at 5 x10^5 cells/well, incubated for 24h and lysed in 300ul of 1% NP-40 lysis buffer. Lysates were then sonicated and spun down at 10,000 g for 10 min at 4 °C. 16ul of lysates were loaded in reduced loading buffer and run at 100V for 2 h. After transferring onto a nitrocellulose membrane, the blot was incubated in blocking buffer diluted in TBS for 1 hour at room temperature. The membrane was then incubated with rabbit anti-TMPRSS2 antibody (Abcam, ab109131) at 1:1000 dilution and mouse anti-GAPDH antibody (Sigma, G8795) at 1:20,000 dilution overnight at 4 °C. The following day, the blot was washed 4 times with TBS-0.1% Tween 20 (TBS-T) and incubated in a mixture of anti-rabbit IRDye 800CW (LI-COR, 926-32211) and anti-mouse IRDye 680RD (LI-COR, 926-68070) antibodies each at 1:2000 dilution for 1 h at room temperature. Finally, the blot was washed 4 times with TBS-T and scanned on LI-COR Biosciences Odyssey Infrared Imaging System with the following specifications: Channel = 800 and 600, Intensity = auto, focal length = 0 mm, resolution = 42 µm.

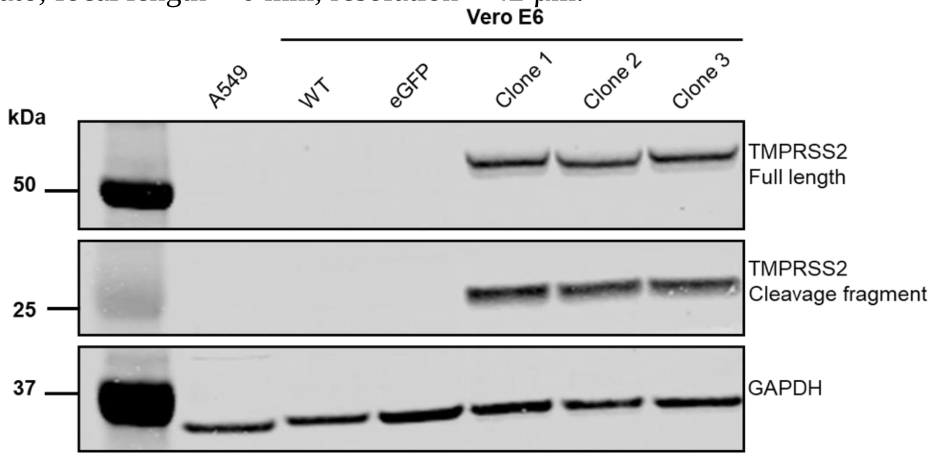

Supplementary Note 1, Figure 1: Western blot to detect TMPRSS2 expression. Lysates from WT, EGFP-expressing and 3 clones of TMPRSS2-expressing Vero E6 cells and A549 cells were used, and the blot was probed with anti-TMPRSS2 or anti-GAPDH antibodies. Clone 1 was used in all further experiments.

Supplementary Note 2

Generation of Norovirus and SARS-CoV-2 linkers

For the generation of both the Norovirus and SARS-CoV-2 linker fragments, a "backbone linker" amplicon of 1061bp containing the a) Hepatitis delta virus ribozyme sequence (HDVr) and SV40 poly A signal for

efficient termination of transcription, b) a spacer of 353bp, and c) Cytomegalovirus (CMV) promoter sequence was first amplified using as a template the flaviUTR-linker[1] and primer pair PUC19flavi_F and CMV_MNV_R (Supplementary Table 3). The backbone amplicon was then joined with the NoV_Ultramer oligonucleotide and extended into a complete linker fragment in a combined reaction with PrimeStar GXL DNA Polymerase (Takara Bio, Japan) using the NoV_Ultramer, the "backbone linker", and primers Ultramer_F and MNV_Link.R (Supplementary Table 3). The following thermocycling conditions were used: 98°C for 30 seconds followed by 5 amplification cycles of 98°C for 10 seconds, 68°C for 20 seconds, 68°C for 90 seconds, and another 30 amplification cycles of 98°C for 10 seconds, 68°C for 20 seconds, 68°C for 90 seconds. For further convenience, the resulted complete MNV-UTR-linker fragment (1182bp) was cloned into a pUC19 vector. The plasmid containing the complete MNV-UTR-linker fragment was amplified in *E. coli* (DH5a) and confirmed by Sanger sequencing. This pUC-19 MNV linker plasmid was then subsequently used as a template to generate HuNoV linker fragment using primers HuNoV_Linker.F and HuNoV_Linker.R (Supplementary Table 3) and SARS-CoV-2 linker fragment using primers CoV-Linker_F and CoV-Linker_R (Supplementary Table 3).

Supplementary Note 3

Generation of Ross River virus CMV and OpiE2 linkers

CMV linker. Firstly, an RRV backbone fragment of 1078 bp containing the HDVr, SV40 poly (A) signal, CMV promoter and the first 22 nucleotides of the RRV 5' UTR region was created using as a template the flaviUTR-linker, primers RRV-Linker-no poly A-F and RRV-Linker-no poly A-R (Supplementary Table 3) and Q5 High-Fidelity DNA Polymerase (New England Bio Lab, USA). The following thermocycling conditions were used: 98°C for 2 minutes followed by 3 amplification cycles of 98°C for 15 seconds, 45°C for 30 seconds, 68°C for 1 minute, then followed by another 32 amplification cycles of 98°C for 15 seconds, 65°C for 30 seconds, 68°C for 1 minute.

Secondly, an RRV-Poly A tail fragment containing 63 adenines was created by using a 134 nucleotide-long RRV_Ultramer oligonucleotide (Supplementary Table 3). To generate a double-stranded DNA product, an

RRV_Ultramer oligonucleotide was used as a template for a PCR reaction using the primers RRV-Poly A-F and RRV-Poly A-R (Supplementary Table 3) to generate RRV-Poly A fragment.

Finally, an RRV backbone fragment was fused with RRV-Poly A fragment by a fusion-PCR to generate CMV-Alpha-UTR-Linker fragment. This was done in 2 steps. In the first step (without primers), both fragments were mixed using equimolar amounts of DNA and subjected to a PCR reaction following the thermocycling conditions: 98°C for 2 minutes followed by 10 amplification cycles of 98°C for 15 seconds, 60°C for 30 seconds, 68°C for 2 minutes. In the second step, 3uL of the PCR product from step 1 was used as a template to amplify the full alpha-UTR-linker fragment by using primers Alpha-UTR-Linker-F and Alpha-UTR-Linker-R (Supplementary Table 3). The thermocycling conditions were: 98°C for 2 min followed by 35 amplification cycles of 98°C for 15 sec, 60°C for 30 sec, 68°C for 2 min. The final fragment CMV-Alpha-UTR-Linker is 1188 bp long and contains the following elements: a) the last 47 nucleotides of the RRV 3'UTR, b) RRV poly-A tail that includes 63 adenines, c) HDVr, d) a spacer sequence of 353bp, e) CMV promoter, and f) the first 22 nucleotides of the RRV 5' UTR region (Fig 3a). For further convenience, it was cloned into a pUC19 plasmid vector. The plasmid containing the CMV-Alpha-UTR-linker fragment was amplified in *E. coli* (DH5a) and confirmed by Sanger sequencing.

OpIE2 linker. To generate a construct capable of producing infectious virus in insect cells, we generated a linker fragment containing a previously optimised *Orgyia pseudotsugata multicapsid nucleopolyhedrovirus* immediate-early 2 (OpIE2) promoter for insect cell expression[2]. Firstly, an OpIE2-linker amplicon of 980 bp containing the HDVr, SV40 poly (A) signal, the OpIE2 promoter, and the first 22 nucleotides of the RRV 5' UTR was amplified from previously described flavivirus OpIE2-linker[2] using primers RRV-Linker-F and OpIE2-RRV-R (Supplementary Table 3) and Prime Star GXL DNA polymerase (Takara Bio, Japan). Secondly, a 134 nucleotide-long RRV_Ultramer oligonucleotide containing the last 47 nucleotides of the viral 3'UTR, the RRV-Poly A tail, and the first 24 nucleotides of the HDVr was converted to dsDNA by PCR using primers RRV-Poly A-F and RRV-Poly A-R (Supplementary Table 3). This amplicon was then joined with the OpIE2 linker amplicon by overlap-extension PCR using GXL DNA polymerase and the flanking primers

RRV-Poly A-F and OpIE2-RRV-R to produce the final 1090bp long OpIE2-Alpha-UTR-Linker fragment (Supplementary Table 3).

Supplementary Note 4

Generation of a CASV OpIE2-linker

Firstly, an OpIE2-linker amplicon of 979bp containing the HDVr, SV40 poly (A) signal, the OpIE2 promoter, and the first 25 nucleotides of the CASV 5' UTR region was amplified using primers HDVr_F and CASV 5' linker R (Supplementary Table 3) from a previously described OpIE2 flavivirus linker template[2]. Following this, a 117 nucleotide-long CASV Ultramer oligonucleotide (Supplementary Table 3) was converted to double-stranded DNA by PCR with primers CASV 3'UTR poly AF2 and RRV-Poly A-R (Supplementary Table 3). These two PCR products combined in equimolar amounts in a final overlap-extension PCR with the flanking primers CASV 3'UTR poly AF2 and CASV 5' linker R (Supplementary Table 3) using PrimeStar GXL DNA polymerase (Takara Bio, Japan). This reaction generated a final 1072bp long CASV OpIE2 linker fragment containing: a) the last 40 nucleotides of CASV 3'UTR, b) the poly-A tail with 54 adenines b) an HDVr, c) a spacer sequence, d) OpIE2 promoter[2], and e) the first 25 nucleotides of the CASV 5'UTR sequence.

References

1 Setoh, Y. X. *et al.* Systematic analysis of viral genes responsible for differential virulence between American and Australian West Nile virus strains. *J Gen Virol* **96**, 1297-1308, doi:10.1099/vir.0.000069 (2015).

2 Piyasena, T. B. H. *et al.* Infectious DNAs derived from insect-specific flavivirus genomes enable identification of pre- and post-entry host restrictions in vertebrate cells. *Sci Rep* **7**, 2940, doi:10.1038/s41598-017-03120-1 (2017).
