# Supplementary material for: A versatile reverse genetics platform for SARS-CoV-2 and other positive-strand RNA viruses
Source: Nat Commun. 2021 Jun 8;12:3431. doi: 10.1038/s41467-021-23779-5 (PMC8187723; doi:10.1038/s41467-021-23779-5)
Supplement: Supplementary file 3 — Reporting Summary [file 41467_2021_23779_MOESM3_ESM.pdf]

nature research

Corresponding author(s): Alexander Khromykh

Last updated by author(s): 29/4/2021

Reporting Summary

Nature Research wishes to improve the reproducibility of the work that we publish. This form provides structure for consistency and transparency in reporting. For further information on Nature Research policies, see our Editorial Policies and the Editorial Policy Checklist.

Statistics

For all statistical analyses, confirm that the following items are present in the figure legend, table legend, main text, or Methods section.

n/a	Confirmed	
☐	☒	The exact sample size (n) for each experimental group/condition, given as a discrete number and unit of measurement
☐	☒	A statement on whether measurements were taken from distinct samples or whether the same sample was measured repeatedly
☐	☒	The statistical test(s) used AND whether they are one- or two-sided<br>*Only common tests should be described solely by name; describe more complex techniques in the Methods section.*
☐	☒	A description of all covariates tested
☐	☒	A description of any assumptions or corrections, such as tests of normality and adjustment for multiple comparisons
☐	☒	A full description of the statistical parameters including central tendency (e.g. means) or other basic estimates (e.g. regression coefficient) AND variation (e.g. standard deviation) or associated estimates of uncertainty (e.g. confidence intervals)
☐	☒	For null hypothesis testing, the test statistic (e.g. F, t, r) with confidence intervals, effect sizes, degrees of freedom and P value noted<br>*Give P values as exact values whenever suitable.*
☒	☐	For Bayesian analysis, information on the choice of priors and Markov chain Monte Carlo settings
☒	☐	For hierarchical and complex designs, identification of the appropriate level for tests and full reporting of outcomes
☐	☒	Estimates of effect sizes (e.g. Cohen's d, Pearson's r), indicating how they were calculated

Our web collection on statistics for biologists contains articles on many of the points above.

Software and code

Policy information about availability of computer code

Data collection	For Flow Cytometry: Data was analyzed using Flowjo v10.<br>For Histology and Scoring images extracted using Aperio ImageScope software v12.3.2.8013 (Leica Biosystems, Wetzlar, Germany). Automatic quantitation of nuclei count and whitespace of H&E stained sections was undertaken using QuPath v0.2.3 (https://qupath.github.io/)<br>Image acquisition of plates for SARS-CoV-2 plaque assays was undertaken using Image Studio Lite (v 5.2.5)
Data analysis	For Bioinformatics analysis: Base-calling and adapter trimming of fast5 files were performed using the guppy_basecaller (Version: 3.3.0 +ef22818). Clean fastq base called files were subsequently mapped to the SARS-CoV-2 isolate QLD02 (GISAID accession EPI_ISL_407896) using the Burrows-Wheeler Aligner (Version: 0.7.13-r1126) using the flags (mem -x ont2d) . Depth of coverage of mapped alignment files was determined using samtools (v1.3) depth. Single Nucleotide Variants (SNVs) of alignment files were identified using iVar (v1.2.2) with a minimum quality score threshold of 20. Coverage and frequencies of variant positions were visualized and calculated using Integrative Genomics Viewer (Version: 2.7.0). For statistical analysis GraphPad Prism (v9.0.0) was used.

For manuscripts utilizing custom algorithms or software that are central to the research but not yet described in published literature, software must be made available to editors and reviewers. We strongly encourage code deposition in a community repository (e.g. GitHub). See the Nature Research guidelines for submitting code & software for further information.

Data

Policy information about availability of data

All manuscripts must include a data availability statement. This statement should provide the following information, where applicable:

- Accession codes, unique identifiers, or web links for publicly available datasets
- A list of figures that have associated raw data
- A description of any restrictions on data availability

Data availability statement
The SARS-CoV-2 QLD02 P3, P4 and CPER cDNA consensus sequence has been deposited in GenBank with the primary accession code MW772455 (http://www.ncbi.nlm.nih.gov/nuccore/MW772455). Raw sequencing data generated in this study are available in the Sequence Read Archive hosted by the National Center for Biotechnology Information with accession number PRJNA707404 (https://www.ncbi.nlm.nih.gov/bioproject/PRJNA707404). Raw data underlying the results shown in Figs. 1d, e, 2e,jm, 3d,g,h, 4c,f, supplementary Figs. 2b,3e-f and 4b-d are provided in Source Data file. The authors declare that all other data supporting the findings of this study are available within the paper, its supplementary information files or the source data file provided with this paper.

Field-specific reporting

Please select the one below that is the best fit for your research. If you are not sure, read the appropriate sections before making your selection.

- [x] Life sciences
- [ ] Behavioural & social sciences
- [ ] Ecological, evolutionary & environmental sciences

For a reference copy of the document with all sections, see nature.com/documents/nr-reporting-summary-flat.pdf

Life sciences study design

All studies must disclose on these points even when the disclosure is negative.

Sample size	Sample size was estimated using G*Power package
Data exclusions	No data was excluded
Replication	Replication was performed as indicated in the methods and figure legends. In vitro experiments were repeated at least twice with similar results unless otherwise indicated in the figure legends. In vivo experiments were performed with the indicated number of mice.
Randomization	Randomization is not applicable for in vitro experiments as cells with different treatments cannot be randomized.
Blinding	Investigators were not blinded during experiments, particularly as animal experiments required infection with SARS-CoV-2 and CPER SARS-CoV-2 and treatment groups needed to be clear to perform the appropriate treatments. Experiments were designed with the indicated controls, and samples for comparison were collected and analyzed under the same conditions.

Reporting for specific materials, systems and methods

We require information from authors about some types of materials, experimental systems and methods used in many studies. Here, indicate whether each material, system or method listed is relevant to your study. If you are not sure if a list item applies to your research, read the appropriate section before selecting a response.

Materials & experimental systems

n/a	Involved in the study	
☐	☒	Antibodies
☐	☒	Eukaryotic cell lines
☒	☐	Palaeontology and archaeology
☐	☒	Animals and other organisms
☒	☐	Human research participants
☒	☐	Clinical data
☒	☐	Dual use research of concern

Methods

n/a	Involved in the study	
☒	☐	ChIP-seq
☐	☒	Flow cytometry
☒	☐	MRI-based neuroimaging

Antibodies

Antibodies used	Primary:<br>anti-CASV 9D7 (Validated by reference in text for IFA Newton et al., 2020) Preparation of Mouse Antiserum and Monoclonal Antibodies to CASV for Immuno florescence Assay. (Produced by Natalee Newton, Jody-Hobson Peters and Roy Hall, School of Chemistry and Molecular Biosciences, University of Queensland, 2018)

anti- SARS-CoV Spike protein monoclonal antibody CR3022 (ter Meulen et al., 2006) cross reactive to SARS-CoV-2 for immune plaque assay (iPA) (Validated by reference Amarilla et al., 2021, Produced by Alberto Amarilla and Naphak Modhiran, School of Chemistry and Molecular Biosciences, University of Queensland, 2020)
mouse anti-E1 monoclonal antibody G8 (Produced by Jody-Hobson Peters and Roy Hall, School of Chemistry and Molecular Biosciences, University of Queensland)

Commercial antibodies:
Rabbit anti-human TMPRSS2 antibody (Abcam, ab109131, Clone number: EPR3862, Isotype: IgG) from website: https://www.abcam.com/TMPRSS2-antibody-EPR3862-ab109131.html
IRDye® 800CW Goat anti-Rabbit IgG Secondary Antibody (LI-COR, 926-32211)
From website: https://www.licor.com/bio/reagents/irdye-800cw-goat-anti-rabbit-igg-secondary-antibody
IRDye® 680RD Goat anti-Mouse IgG Secondary Antibody (LI-COR, 926-68070)
From website: https://www.licor.com/bio/reagents/irdye-680rd-goat-anti-mouse-igg-secondary-antibody
IRDye® 800CW Goat anti-Mouse IgG Secondary Antibody (LI-COR, 926-32210)
From website: https://www.licor.com/bio/reagents/irdye-800cw-goat-anti-mouse-igg-secondary-antibody
Monoclonal Anti-GAPDH−Peroxidase antibody produced in mouse (clone GAPDH-71.1, purified from hybridoma cell culture, Sigma, Cat number: G8795) From website: https://www.sigmaaldrich.com/catalog/product/sigma/g9295
mouse anti-MNV1 capsid antibody (Cat: MABF2097 Merk Millipore, Clone: 5C4.10) From website: https://www.sigmaaldrich.com/catalog/product/mm/mabf2097
anti-Mouse HRP secondary antibody (Cat: G-21040 ThermoFisher Scientific, RRID:AB_2536527) From website: https://www.thermofisher.com/antibody/product/Goat-anti-Mouse-IgG-H-L-Cross-Adsorbed-Secondary-Antibody-Polyclonal/G-21040
Anti-Actin antibody produced in rabbit (MDL number MFCD00145889, NACRES NA.41, Merck/Sigma Cat number A2066) From website: https://www.sigmaaldrich.com/catalog/product/sigma/a2066
Donkey anti-Rabbit IgG (H+L) Highly Cross-Adsorbed Secondary Antibody, HRP (RRID: AB_2534709, Catalog # A16035, Thermo Fisher) From website: https://www.thermofisher.com/antibody/product/Donkey-anti-Rabbit-IgG-H-L-Highly-Cross-Adsorbed-Secondary-Antibody-Polyclonal/A16035
Goat anti-Human IgG Fc Highly Cross-Adsorbed Secondary Antibody, HRP (Invitrogen, Thermo Fisher, Cat number: A18829, RRID: AB_2535606) From website: https://www.thermofisher.com/antibody/product/Goat-anti-Human-IgG-Fc-Highly-Cross-Adsorbed-Secondary-Antibody-Polyclonal/A18829

Validation

Validation of anti-CASV 9D7 (Validated by reference Newton et al., 2020)
Preparation of Mouse Antiserum and Monoclonal Antibodies to CASV
Produced by Natalee Newton, Jody-Hobson Peters and Roy Hall, School of Chemistry and Molecular Biosciences, University of Queensland, 2018).
Hybridomas secreting monoclonal antibodies specific to CASV were derived from BALB/c mice immunised with purified virions (Piyasena et al., 2017). Hybridomas were maintained at 37 ◦C 5% CO_2 in hybridoma serum-free media supplemented with 50 µg/mL streptomycin, 50 U/mL penicillin and 2 mM L-glutamine with 20% FBS initially and then weaned to growth in serum-free media. Isotyping of the mAbs was performed using the Mouse Monoclonal Antibody Isotyping Reagent (Sigma Aldrich Australia, Castle Hill, NSW, Australia) as per the manufacturer's instructions. The reactivity of each mAb to CASV antigen was assessed by ELISA as previously described, using fixed CASV infected C6/36 cell monolayers.
Validation of anti- SARS-CoV Spike protein monoclonal antibody CR3022 (ter Meulen et al., 2006) cross reactive to SARS-CoV-2 for immune plaque assay (iPA) (Validated by reference Amarilla et al., 2021,

Produced by Alberto Amarilla and Naphak Modhiran, School of Chemistry and Molecular Biosciences, University of Queensland, 2020
Recombinant monoclonal antibodies (mAb) that recognize SARS-CoV-2, including CR3022 were generated in Chinese hamster ovary (CHO) cells (ter Meulen et al., 2006). Briefly, the variable regions for each of the antibodies were codon-optimized and synthesized for Cricetulus griseus (hamster) cell expression by Integrated DNA Technologies (Singapore) and cloned in-frame into plasmids encoding the human or mouse constant heavy- and light-chain IgG1 backbone as described previously (Jones et al., 2010). The CHO cells were co-transfected with plasmids encoding the heavy and light chain for each antibody, and at 7 days post-transfection, the antibody was purified with a protein A column (GE healthcare). The antibodies were validated by SDS-PAGE and ELISA before use.

Validation of the anti-E1 monoclonal antibody G8
Produced by Jody-Hobson Peters and Roy Hall, School of Chemistry and Molecular Biosciences, University of Queensland.
Mouse immunisation and hybridoma production: BALB/c mice 6–8 weeks of age were immunised intraperitoneally (i.p.) with 5 µg of sucrose gradient-purified Ross River Virus (T48 strain). Two immunisation via i.p. route we performed with 2 weeks period of intervals between each immunisation. A third and final boost was performed intravenously. Mice spleens were harvested at 3 days after the last boost and the spleen B cells were fused with myeloma line PRX63 as previously described (Oliveira et al., 1997). Monoclonal antibody affinity of G8 against RRV-T48 was validated by Western blot, neutralization assay, ELISA and IFA in the following (Oliveira et al., 1997, Oliveira et al., 2006 and Goh, et al., 2013).

REFERENCES
Goh LYH., Hobson-Peters, J., Prow, NA., Gardner, J., Bielefeldt-Ohmann, H., Pyke, AT., Suhrbier, A., Hall R.A., Neutralizing monoclonal antibodies to the E2 protein of chikungunya virus protects against disease in a mouse model. Clin Immunol. 2013 Dec;149(3):487-97.
Jones, M. L., Seldon, T., Smede, M., Linville, A., Chin, D. Y., Barnard, R., et al. (2010). A method for rapid, ligation-independent reformatting of recombinant monoclonal antibodies. J. Immunol. Methods 354, 85–90. doi: 10.1016/j.jim.2010.02.001
Oliveira, N. M., Hall, R., Broom, A., Lindsay, M., Weir, R., Kay, B., & Mackenzie, J. (1997). Epitope analysis of Ross River virus using monoclonal antibodies. Arbovirus Research in Australia, 7, 201-207.
Oliveira, N. M., Broom, A., Mackenzie JA., Smith, DW., Lindsay, MDA., Kay,BH., Hall, RA., Epitope-Blocking Enzyme-Linked Immunosorbent Assay for Detection of Antibodies to Ross River Virus in Vertebrate Sera. Clin Vaccine Immunol. 2006 Jul; 13(7): 814–817.

Piyasena, T.B.H.; Setoh, Y.X.; Hobson-Peters, J.; Newton, N.D.; Bielefeldt-Ohmann, H.; McLean, B.J.; Vet, L.J.; Khromykh, A.A.; Suhrbier, A. Infectious DNAs derived from insect-specific flavivirus genomes enable identification of pre- and post-entry host restrictions in vertebrate cells. Sci. Rep. 2017, 7, 1–11.
ter Meulen, J., van den Brink, E. N., Poon, L. L., Marissen, W. E., Leung, C. S., Cox, F., et al. (2006). Human monoclonal antibody combination against SARS coronavirus: synergy and coverage of escape mutants. PLoS Med. 3:e237. doi: 10.1371/journal.pmed.0030237

Eukaryotic cell lines

Policy information about cell lines

Cell line source(s)	Aedes albopictus C6/36 (ATCC® CRL-1660)<br>Murine fibroblast NIH3T3 cells (ATCC® CRL-1658™)<br>Murine macrophage RAW264.7 (ATCC® number TIB-71)<br>Cercopithecus aethiops Vero E6 (Vero 76, clone E6, ATCC CRL-1568)<br>Human embryonic kidney 293T cells (ATCC® CRL-3216™)<br>HEK293T-hACE2 cells were provided by Jesse Bloom (Fred Hutchinson Cancer Research Center, Washington, USA) originating from Human embryonic kidney 293T cells (ATCC, CRL-3216).
Authentication	Other cell lines were not genetically confirmed, but their morphologies were visually confirmed.<br>Production of TMPRSS2 expressing VeroE6 cell lines (VeroE6-TMPRSS2) and validation have been provided in Supplementary Note 1.
Mycoplasma contamination	All cells were tested and confirmed mycoplasma-negative
Commonly misidentified lines<br>(See ICLAC register)	No commonly misidentified cell lines were used in this study.

Animals and other organisms

Policy information about studies involving animals; ARRIVE guidelines recommended for reporting animal research

Laboratory animals	Heterozygous K-18 hACE2-transgenic mice (The Jackson Laboratory, Bar Harbour, ME, USA) were bred in-house by crossing with C57BL/6J mice (male and female mice 8 weeks old) (Animal Resources Center, Canning Vale, WA, Australia). DNA from the tail was isolated using Extract-N-Amp Tissue PCR kit (Sigma) and PCR genotyping undertaken as described (The Jackson Laboratory. Genotyping protocols database. B6.Cg-Tg(K18-ACE2)2Prlmn/J. Stock No: 034860. Protocol 38276), except using primers Forward – 5'-CTTGGTGATATGTGGGGTAGA-3', reverse 5' CGCTTCATCTCCCACCACTT-3' (recommended by NIOBIOHN, Osaka, Japan). 6-8 week old male K-18 hACE2 mice (n=3 per group) were used.
Wild animals	No wild animals were used for this study.
Field-collected samples	No field collections were used for this study.
Ethics oversight	Mouse experiments were approved by the QIMR Berghofer MRI Biosafety Committee and Animal Ethics Committee (project P3600) and conducted in accordance with the "Australian Code for the care and use of animals for scientific purposes" as defined by the National Health and Medical Research Council of Australia.

Note that full information on the approval of the study protocol must also be provided in the manuscript.

Flow Cytometry

Plots

Confirm that:

- [x] The axis labels state the marker and fluorochrome used (e.g. CD4-FITC).
- [x] The axis scales are clearly visible. Include numbers along axes only for bottom left plot of group (a 'group' is an analysis of identical markers).
- [x] All plots are contour plots with outliers or pseudocolor plots.
- [x] A numerical value for number of cells or percentage (with statistics) is provided.

Methodology

Sample preparation	Cells used for flow cytometry analysis (ZsGreen expression) were washed with PBS, treated with 500uL of trypsin for 5min at 37oC, spun at 500g for 5 min and resuspended in 500uL of DMEM (containing 2% FCS and P/S). Cells were fixed with 4% paraformaldehyde (PFA) for 30 min. To remove the PFA, cells were spun down at 500g for 5 min and resuspended in 1mL of the ice-cold PBS containing 5% heat-inactivated FCS once. Lastly, cells were spun down at 500g for 5 min and then resuspended in ice-cold PBS and passed through 70 µm mesh filter prior to analysis.
Instrument	BD AccuriTM C6 analysis.

Software	Flowjo v10.
Cell population abundance	10,000 events in P1 for Mock (uninfected) and ZsGreen infected
Gating strategy	Ten thousand cells were gated using Side Scatter (SSC-A) verses Forward Scatter (FSC-A), single cells were selected using Forward Scatter (FSC-H) vs Forward Scatter (FSC-A). Fluorescence was measured through a 424/44 bandpass filter in front of fluorescence 1 (FL1) detector. Single cells were then analysed for median fluorescence intensity (MFI) Relative fluorescence intensities, which corresponded to relative zsGreen content of gated populations, were acquired on histograms of FL1 pulse area (FL1-A).

☒ Tick this box to confirm that a figure exemplifying the gating strategy is provided in the Supplementary Information.
